# Muscle Excitability Scale for the assessment of spastic reflexes in spinal cord injury: development and evaluation

**DOI:** 10.1038/s41393-024-01016-2

**Published:** 2024-07-17

**Authors:** Jiri Kriz, Zuzana Nasincova, Veronika Gallusova, Tomas Vyskocil, Martin Gregor, Krystof Slaby, Kristyna Sediva

**Affiliations:** 1 Spinal Cord Unit, Department of Rehabilitation and Sports Medicine, 2nd Faculty of Medicine, Charles University and University Hospital Motol, Prague, Czech Republic; 2 Department of Rehabilitation and Sports Medicine, 2nd Faculty of Medicine, Charles University and University Hospital Motol, Prague, Czech Republic; 3 Paraple Centre, Prague, Czech Republic

**Keywords:** Spinal cord diseases, Motor neuron disease

## Abstract

**Study design:**

A psychometric study.

**Objectives:**

To introduce a novel simple tool designed to evaluate the intensity of the phasic (dynamic) component of spastic motor behavior in spinal cord injury (SCI) people and to assess its reliability and validity.

**Setting:**

The study was developed in the Spinal Cord Unit at University Hospital Motol and Paraple Centre in Prague, Czech Republic.

**Methods:**

The Muscle Excitability Scale (MES) is designed to rate muscle motor response to exteroceptive and proprioceptive stimuli. The impairment rating ranges from zero muscle/muscle group spasm or clonus to generalized spastic response. The selected 0 to 4 scale allows for comparing the MES results with those of the Modified Ashworth Scale (MAS). After long-term use and repeated revisions, a psychometric analysis was conducted. According to the algorithm, two physiotherapists examined 50 individuals in the chronic stage after SCI.

**Results:**

The inter-rater reliability of MES for both legs showed *κ* = 0.52. The intra-rater reliability of MES for both legs showed *κ* = 0.50. The inter-rater reliability of simultaneously assessed MAS for both legs was higher, with *κ* = 0.69. The intra-rater reliability of MAS for both legs showed *κ* = 0.72. Spearman’s rank correlation coefficient between MES and spasm frequency of Penn Spasm Frequency Scale (PSFS) was low, while the correlation coefficient between MES and the severity part of PSFS was moderate.

**Conclusions:**

The MES is a complementary tool for assessing the dynamic component of spastic motor behavior in SCI people. It allows a more comprehensive clinical characterization of spastic reflexes when used along with the MAS.

## Introduction

In people following spinal cord injury (SCI), spastic motor behavior is defined primarily by hypertonia, flexor and extensor spasms, and clonus. Another characteristic feature, especially in those with complete SCI, is segmental or general spasticity which requires a different approach than focal spasticity in people following brain injury [1]. Therefore, the primary goal in people with SCI is to gain a general perspective of spastic motor behavior rather than of the spasticity of individual muscles.

From a diagnostic and therapeutic perspective, it is meaningful to divide spastic motor behavior into tonic (static) and phasic (dynamic) components or patterns [2]. While the tonic component is represented by muscle hypertonia (spasticity in the narrower sense), the phasic component primarily encompasses flexor and extensor spasms and clonus. These components are also known as hypokinetic and hyperkinetic types [3] and have varying effects on the individual’s physical and psychological well-being. In the post-acute stage, after the spinal shock has subsided, spastic motor behavior gradually increases, often up to the point where it significantly negatively influences the training of some activities of daily living (ADL), causes pain, or disturbs sleep. Physiotherapeutic methods have a limited and short-term effect on reducing both components and drug therapy needs to be initiated. Our longstanding clinical experience directs to the fact that each component is modifiable by a specific type of medication. In people with a predominance of the tonic component, the drug of first choice is usually baclofen, while the phasic component is more responsive to gabapentinoids. Despite the repeated use of gabapentinoids for spasticity or spasms, their therapeutic effects have not been further specified [4, 5].

In order to choose the appropriate medication and monitor its efficacy, we needed a simple clinical examination that could distinguish between the two components of spastic motor behavior and measure their intensity. To assess muscle hypertonia, we utilized the Modified Ashworth Scale (MAS) [6]. Although there are conflicting results regarding the reliability of the MAS for evaluating spasticity in the lower limbs, it remains the most widely used assessment scale for SCI patients. However, no similar scale is available to evaluate the phasic component. The only options are a subjective Penn Spasm Frequency Scale [7] or a three-domain instrument, the Spinal Cord Assessment Tool for Spastic Reflexes [8]. To address this limitation, we have developed the Muscle Excitability Scale (MES), which provides a more comprehensive evaluation of the phasic (dynamic) component. Differentiating and quantifying the overall tonic and phasic response can facilitate selecting appropriate pharmacotherapy and evaluating its effectiveness.

## Methods

A Muscle Excitability Scale (MES) was designed and has long been used by physicians and physiotherapists at the Spinal Cord Unit of the University Hospital Motol in Prague, Czech Republic. After the methodology was established, a pilot reliability study was conducted on 49 SCI people by undergraduate students of the Second Faculty of Medicine, Charles University, within their master’s degree theses. Because of disproportional grade distribution, the scale needed to be revised, and the definitions of motor responses to stimuli were refined and clarified for each grade. A study to assess the inter-rater and intra-rater reliability and convergent validity of the scale was then performed (ClinicalTrials.gov Identifier: NCT04266964).

### Instrument

The principle of the Muscle Excitability Scale is to quantify muscle motor response (muscle tendency to spasms or clonus) to exteroceptive and proprioceptive stimuli (Table 1).

**Table 1:** Muscle Excitability Scale (MES).

Grade	Description
0	No motor response (muscle spasm or clonus) to a skinfold squeeze or passive movement
1	Motor response to a skinfold squeeze OR passive movement
2	Motor response to both a skinfold squeeze AND passive movement
3	Strong motor response to a skinfold squeeze AND/OR passive movement
4	Generalized motor response to a skinfold squeeze AND/OR passive movement

#### Examination algorithm

The individual is placed in the supine position, i.e., lying relaxed on the back with the lower and upper extremities extended, possibly with the head rested on a pad. Before the examination, the individual should remain in this position for at least five minutes.

**Fig. 1:**
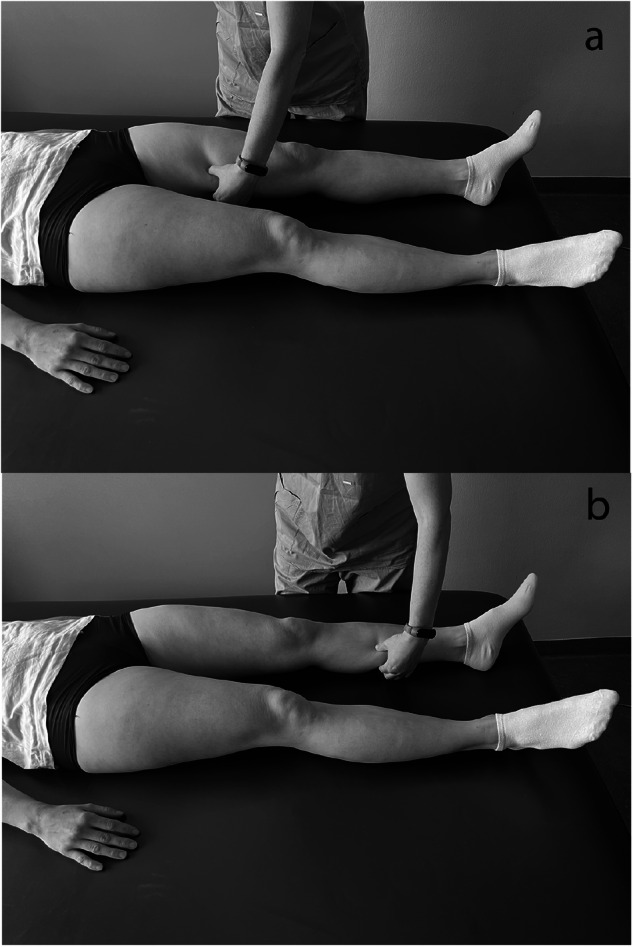
Exteroceptive stimuli. The examiner squeezes the skinfold at the inner side of the middle thigh (**a**) and at the inner side of the middle calf (**b**).

Step 1: The examiner squeezes the skinfold with the thumb and index finger once at the inner side of the middle thigh and once at the inner side of the middle calf (Fig. 1).

**Fig. 2:**
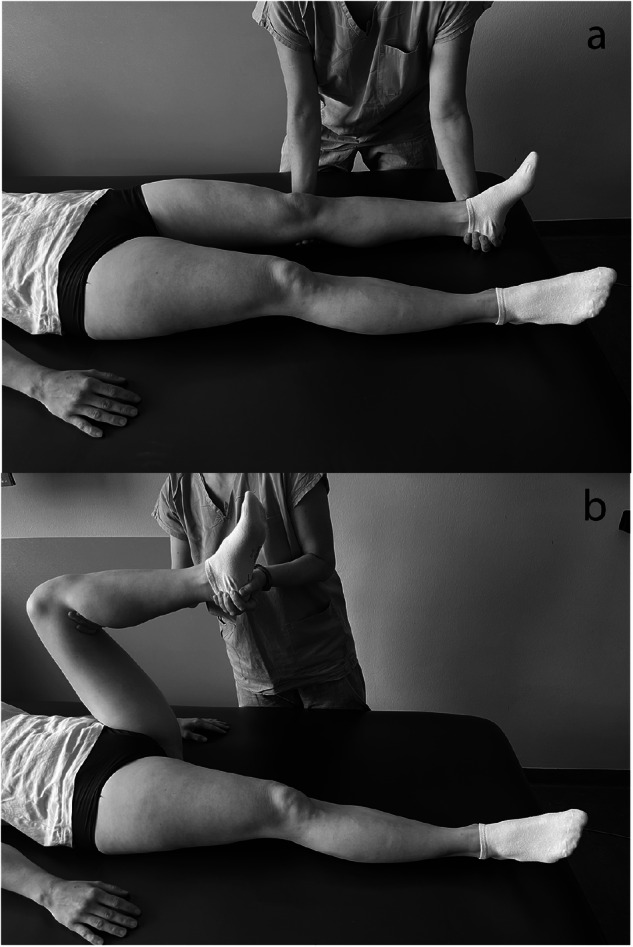
Proprioceptive stimuli. The examiner places his hand under the knee and the heel (**a**) and moves the leg into maximum flexion at the hip and knee joints (**b**), and then returning it to full extension.

Step 2: Following that, he places his hand under the knee and heel and moves the leg into maximum flexion at the hip and knee joints. After a response, if any, the examiner moves the limb back into full extension (Fig. 2). Each of these movements lasts for one second. In the evaluation, the more significant reaction to these two maneuvers is taken into account.

#### Rating algorithm

MES 0—neither a skinfold squeeze nor a passive movement into full flexion and subsequent extension at the hip and knee joints causes a visible muscle spasm or clonus in the lower limb.

MES 1—skinfold squeeze at the thigh and/or calf causes a muscle spasm (jerk), or a muscle jerk is caused by the subsequent passive movement (not resistance as in the case in hypertonia, but rather an additional movement).

MES 2—both stimuli (skinfold squeeze and passive movement) cause a muscle jerk manifested mainly by a discreet movement of the lower limb (in the case of skinfold squeeze, e.g., by lower limb external or internal rotation or acral movements).

MES 3—skinfold squeeze is usually followed by hip and knee flexion, which is greater than one-third of the range of motion, and/or passive movement causes intensive additional motor response on the lower limb (withdrawal of the leg from the rater’s hand). If there is any movement in the other limb, it is less than one-third of the range of motion, usually tending to move into internal or external rotation,

MES 4—apart from the above-mentioned response to both stimuli, the motor response is also transmitted to the other lower limb, where it is greater than one-third of the range of motion. It can also be transmitted to the trunk or upper limbs.

Passive movement into flexion and then into extension at the hip and knee joints applied for one second is consistent with the algorithm that we use in the assessment of the Modified Ashworth Scale. Using a single examination, it is therefore possible to interpret the scores on both scales at the same time. Nevertheless, some level of clinical experience is necessary to clearly differentiate between limited motion due to hypertonia and additional movement due to increased excitability. Videos showcasing examination responses for each grade can be found in the Supplementary file.

### Participants

A total of 50 participants, aged 18–68 years, were enrolled in the study. The sample size was planned based on the anticipated recruitment rate and period, which finally lasted 18 months. All participants were clients of the Paraple Centre (a provider of short-term follow-up inpatient rehabilitation) in Prague, Czech Republic, where the examinations were performed. The inclusion criteria were the presence of clinically relevant spasticity of the lower extremities while on stable medication. The exclusion criteria were significant complications that affect spasticity (such as decubitus ulcers, heterotopic ossification, urinary tract infections, or any other infections), and severe limitation in the range of motion (ROM) of the lower extremities.

### Raters

Two physiotherapists experienced in treating people with SCI and using spasticity assessment in their daily practice carried out all of these assessments. To ensure optimal standardization of the MAS and MES assessment, they undertook a one-day training session (acquisition of the protocol, examination of 5 subjects under the lecturer’s supervision, and collaborative analysis of the results) on our Spinal Cord Unit before the initiation of the study. The raters did not discuss testing procedures, outcomes, or other study-related issues during proper data collection. Ratings were performed at the same time of the day. While the individual was lying supine before the examination, the first rater collected the data on the frequency and severity of spasms according to the Penn Spasm Frequency Scale (PSFS). Then, the rater started to examine the right leg first. The time window between the raters’ examinations was ten minutes. One week later, one of the raters repeated the examinations to allow intra-rater reliability to be determined.

### Statistical analysis

Statistical analyses were performed using the Statistica 13.3 software (TIBCO Software Inc., USA). Descriptive statistics were used to provide demographic and impairment characteristics.

Kappa (*κ*) statistics was chosen as a reliability measure of MES and MAS, as this is recommended as the most appropriate measure. Inter-rater reliability was calculated between the two ratings carried out on the same day by different raters unaware of each other’s results. Intra-rater reliability was determined by comparing the outcomes of the same rater obtained one week apart. The interpretation of the strength of agreement of the κ-values is based on Landis and Koch [9].

Spearman’s rank correlation coefficient was used to identify the relationship between the MES and PSFS scores, with interpretation based on Schober et al. [10].

## Results

### Cohort data

The mean age of the study participants was 39 ± 12 years. Forty-two were males and eight were females. Time since injury ranged from 10 months to 32.4 years. The most common cause of SCI was a traffic accident (30%). Thirty-five participants had a sensorimotor complete injury. Forty-two people regularly received antispastic or antiepileptic medication. The characteristics of the study population are presented in Table 2. The individual participant data can be found in the Supplementary file.

**Table 2:** Study population characteristics.

	Ø	SD	median	IQR
Age (years)	36.4	12.0	36.7	27.4–44.3
Time from SCI (years)	8.4	7.6	6.3	1.9–15.4
	*N*	%		
Gender				
Male	42	84.0		
Female	8	16.0		
Etiology of SCI				
Fall	14	28.0		
Traffic accident	15	30.0		
Sports injury	10	20.0		
Diving into water	9	18.0		
Violence	1	2.0		
Infection	1	2.0		
NLI				
Cervical	35	70.0		
Thoracic	15	30.0		
AIS				
A = Sensorimotor complete	35	70.0		
B = Sensory incomplete	14	28.0		
C = Motor incomplete	1	2.0		
Medication				
Antispastic	26	52.0		
Antiepileptic	16	32.0		
Total	50	100.0		

*SCI* spinal cord injury, *NLI* neurological level of injury, *AIS* American Spinal Injury Association Impairment Scale, *IQR* interquartile range.

**Fig. 3:**
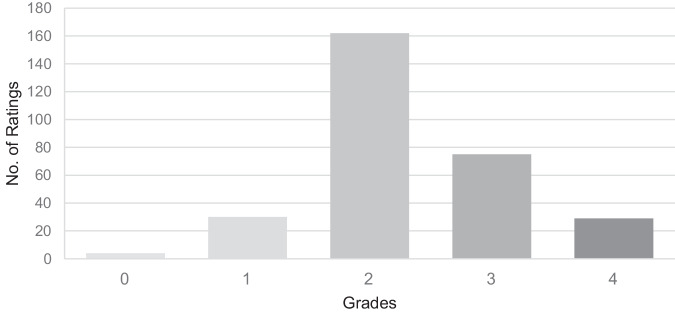
Distribution of MES values. The figure shows the distribution of all grades of Muscle Excitability Scale.

**Fig. 4:**
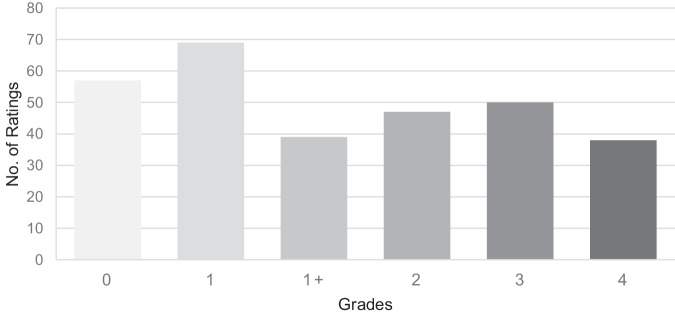
Distribution of MAS values. The figure shows the distribution of all grades of Modified Ashworth Scale.

The distribution of MES and MAS scores is shown in Figs. 3 and 4, respectively.

### MES and MAS reliability

The inter-rater reliability of MES showed weighted *κ* = 0.51 for the right leg and weighted *κ* = 0.54 for the left leg. The intra-rater reliability showed weighted *κ* = 0.41 for the right leg and weighted *κ* = 0.60 for the left leg. The weighted kappa for both legs was interpreted as moderate.

The inter-rater weighted kappa coefficient for MAS was 0.65 for the right leg and 0.73 for the left leg, indicating substantial agreement. The intra-rater agreement measures for MAS were slightly higher than those for inter-rater agreement, and the kappa values were 0.73 and 0.69 for the right and left leg, respectively.

The inter-rater and intra-rater kappa values for MES and MAS are shown in Table 3.

**Table 3:** MES and MAS inter-rater and intra-rater reliability.

	*n*	*κ*-quadratic weight	Interpretation
Muscle Excitability Scale (MES)			
Inter-rater			
Right lower extremity	50	0.507	Moderate*
Left lower extremity	50	0.544	Moderate*
Both lower extremities	100	0.524	Moderate*
Intra-rater			
Right lower extremity	50	0.413	Moderate*
Left lower extremity	50	0.597	Moderate*
Both lower extremities	100	0.503	Moderate*
Modified Ashworth Scale (MAS)			
Inter-rater			
Right lower extremity	50	0.654	Substantial*
Left lower extremity	50	0.726	Substantial*
Both lower extremities	100	0.688	Substantial*
Intra-rater			
Right lower extremity	50	0.734	Substantial*
Left lower extremity	50	0.693	Substantial*
Both lower extremities	100	0.715	Substantial*

*MES* Muscle Excitability Scale, *MAS* Modified Ashworth Scale.

*Significant at *p* < 0.001.

### Correlation between MES and PSFS

To correlate the Muscle Excitability Scale (MES) and Penn Spasm Frequency Scale (PSFS), we only needed to compare two values. Therefore, we took the higher scores from both legs into account from each rater. We correlated the MES data with the frequency and severity parts of PSFS alone. The Spearman’s rank correlation coefficient between MES and the frequency part of PSFS was weak, while the correlation coefficient between MES and the severity part of PSFS was moderate (Table 4).

**Table 4:** Correlations between MES and PSFS.

	Rater	*n*	Spearman *R*	Interpretation
MES max vs. PSFS frequency	R1	50	0.253	Weak
	R2	50	0.389	Weak
MES max vs. PSFS severity	R1	50	0.464	Moderate*
	R2	50	0.527	Moderate*

*MES* Muscle Excitability Scale, *PSFS* Penn Spasm Frequency Scale.

*Significant at *p* < 0.001.

## Discussion

The quantification of the tonic and phasic components is of utmost importance in the post-acute stage of SCI for both the choice of pharmacotherapy and monitoring its effectiveness. A variety of spastic patterns is often observed in the clinical setting with contradictory results obtained for the two components. For instance, an individual with a predominance of hypertonia has his lower limbs stiffened, difficult to stretch or bend, and handling them is not easily accomplished. It limits his/her mobility and ability to perform ADL. On the other hand, an individual with a predominant phasic (dynamic) component of spasticity has highly excitable limbs, the handling of which is not limited by muscle hypertonia but can be associated with sudden rapid spontaneous movements that may increase the risk of falling during ADL. Moreover, spasms and clonus can interfere with the ability to relax while resting, thus contributing to the individual’s overall physical and psychological discomfort. In the chronic stage, the individual partly adapts to the spasticity and the quantification of the presentation in the sense of medication adjustment becomes less relevant. However, the assessment is important in case of a change in health status as well as for clinical trials.

Our goal in developing the Muscle Excitability Scale (MES) to assess the phasic component of spastic motor behavior, was to align it with the Modified Ashworth Scale (MAS), which focuses on the tonic component [11]. The reliability of the MAS in the SCI population has been repeatedly evaluated with various results. For example, Haas et al. found only fair inter-rater reliability between a physiotherapist and a physician when assessing different lower limb muscle groups in 30 individuals with SCI [12]. Craven and Morris concluded that MAS has inadequate inter-rater and inter-temporal reliability in assessing lower limb spasticity [13]. On the other hand, Baunsgaard et al. found satisfactory reliability of MAS in 31 participants using weighted kappa [14]. In a study by Akpinar et al., the reliability of the Modified Ashworth Scale (MAS) and the Modified Tardieu Scale (MTS) was compared in a group of 65 SCI individuals. The study found that both inter-rater and intra-rater reliability for the MAS was moderate to substantial. Regarding the MTS, the quality of the muscle reaction component showed substantial agreement, whereas the angle of muscle reaction (R2-R1) component demonstrated excellent reliability [15]. It is important to note that the R2-R1 component makes the MTS an effective tool for distinguishing between spasticity and contracture in a specific muscle, a crucial aspect in spastic paresis after stroke [16]. However, in individuals with SCI, spasticity tends to be segmental or generalized, requiring a more comprehensive evaluation. Therefore, instead of evaluating MAS for individual muscles, we deliberately simplified the examination to passive flexion in the hip and knee and subsequent extension for each leg. We found substantial inter-rater and intra-rater reliability for these MAS results of both lower limbs in our group. In the context of other studies, this suggests that we used a well-standardized investigative procedure and well-trained, experienced raters.

The MES developed by our team allows for a comprehensive evaluation of dynamic component of spastic motor behavior, i.e., flexion and extension spasms and clonus at the same time. Our deliberate choice encompassed exteroceptive and proprioceptive stimuli to evoke all three dynamic motor responses. Studies have demonstrated that tactile and proprioceptive stimuli can trigger flexion spasms [17, 18] and clonus [19]. Additionally, extension spasms can be induced by proprioceptive input from the hip or knee [20, 21]. Previously, the only clinical evaluation of the dynamic component was presented by Benz et al., which they called the Spinal Cord Assessment Tool for Spastic Reflexes (SCATS). This tool has the disadvantage of needing to involve three different tests which yield three different scores ranging from 0 to 3 points. Apart from being significantly more time-consuming, the examination provides results that are difficult to compare not only to each other but also to the MAS outcomes. The authors tested the validity of the tool by comparing electromyographic and kinematic measurements and confirmed a significant correlation [8]. Akpinar et al. evaluated the reliability of the SCATS on 47 SCI subjects. Inter-rater and test-retest reliability was substantial to almost perfect [22]. In addition, all parts of the SCATS and MAS tests for different muscles were correlated with each other, which, from our point of view, is unjustified due to the evaluation of different manifestations of spastic motor behavior. Therefore, we deliberately did not perform a correlation between MAS and MES.

Penn et al. designed a scale based on a self-report questionnaire for people after spinal cord injury [7]. Their scale has two parts to assess the frequency and severity of spasms [23]. Mills et al. have tested the reliability of the Penn Spasm Frequency Scale (PSFS) in chronic SCI people and found it “almost perfect” [24]. However, it has the disadvantage of analyzing based on self-reported information, with the individual focusing on spasms associated with specific situations, such as difficulty moving from the wheelchair. Spasm frequency also varies with the individual’s activity level over time. Benz et al. found no correlation between the PSFS and SCATS scores, which may be due to the fact that flexor and extensor spasms are triggered during specific ADL [8].

The advantage of the MES we designed is primarily a rapid and easy-to-perform examination that can be used in daily clinical practice. Together with the MAS, the examiner receives two values that allow him to get a comprehensive picture of spastic motor behavior in the given patient. Inter-rater and intra-rater reliability for MES was estimated as moderate using weighted kappa. These results are consistent with the high variability of muscle excitability based on many variables. Unlike muscle hypertonia which does not change significantly in response to repeated movement during the examination, muscle excitability often responds to repeated stimuli by a sharp decrease in intensity, even for small stimuli. Therefore, we applied the above-mentioned procedure with the individual being examined in the supine position in the bed without any prior adjustment to avoid the induction of spasms. For the same reason, joint ranges of motion need to be examined in advance. We focused solely on passive flexion of the hip and knee, followed by full extension of the leg. The level of resistance provided insight into the muscle hypertonia grade in the examined segments, as per MAS. The simultaneous examination of both components is supported by other authors [13].

We compared the MES outcomes with the subjective PSFS rating. As for spasm frequency, a weak correlation was found between MES and PSFS. These results were unsurprising because spasm frequency depends on daily activities, as mentioned above. When compared for spasm severity, MES and PSFS showed a higher correlation, which was classified as moderate. This aligns with our MES scale’s focus on the intensity of primary response rather than frequency over time.

## Limitations and future work

The muscle excitability assessment shows a disproportionate distribution of grades in favor of grade 2. There are two main reasons for this. Grades 0 and 1 were missing as only individuals who showed signs of muscle excitability were included in the study. The highest prevalence of grade 2 indicates compensated muscle excitability in most people, 26 of whom were on appropriate medical therapy. In contrast, the MAS grades were more evenly distributed with a slight predominance of grades 1 and 0. The representation of grades 3 and 4 was relatively high despite 14 people taking the maximum dose of myorelaxants.

Despite a simple and unequivocal assignment to grades based on the clinical presentation, the MES scores may vary for the same patient. This may be due to the examiner’s skillfulness and experience with tactile contact and handling the patient. These influence both the strength of the exteroceptive stimulus and the way of moving the patient’s limb. Similarly, the level of clinical experience may play a role in motor response assessment. A patient’s muscle excitability may also be influenced by certain factors such as his/her mental condition, fatigue, the weather, preceding activities, etc. These are probably more significant than in the assessment of hypertonia. The response to the first stimulus differs considerably from that to repetitive stimuli. Therefore, it is important to measure the intensity of the phasic component at the beginning of the examination after at least five minutes of relaxation in the supine position.

We believe that the development of MES degree definitions is complete. However, conducting further psychometric studies to verify its effectiveness is important. Subgroup analysis to determine the strength of the MES at different levels and severities of the injury would also be beneficial.

## Conclusion

The Muscle Excitability Scale was designed in response to the need to assess the phasic component of spastic motor behavior in SCI people. This simple tool using a 0–4 scale allows the comparison of the scores with those of the Modified Ashworth Scale. This approach makes it possible to obtain a comprehensive evaluation of a patient’s spastic motor behavior and, based on the specific result from the examination, to prescribe individually tailored medication. Currently, we are preparing a double-blinded multicenter study to validate this different effect. The availability of two assessment scales should benefit the individual receiving more effective treatment and open new possibilities for clinical trials addressing this issue.

### Supplementary information

Online supplement

Video 1

Video 2

Video 3

Video 4

Video 5

## Data Availability

The data supporting the findings of this study are available as Supplementary Data.
