# Supplementary material for: Muscle Excitability Scale for the assessment of spastic reflexes in spinal cord injury: development and evaluation
Source: Spinal Cord. 2024 Jul 17;62(9):532–8. doi: 10.1038/s41393-024-01016-2 (PMC11368810; doi:10.1038/s41393-024-01016-2)
Supplement: Supplementary file 1 — Online supplement [file 41393_2024_1016_MOESM1_ESM.docx]

**Online supplement**

**Title:** Muscle Excitability Scale for the Assessment of Spastic Reflexes in Spinal Cord Injury: Development and Evaluation

**Authors:**

Jiri Kriz^1^, Zuzana Hlinkova^2^, Veronika Gallusova^2^, Tomas Vyskocil^3^, Martin Gregor^3^, Krystof Slaby^2^, Kristyna Sediva^2^

**Affiliations:**

^1^Spinal Cord Unit, Department of Rehabilitation and Sports Medicine, 2^nd^ Faculty of Medicine, Charles University and University Hospital Motol, Prague, Czech Republic;

^2^Department of Rehabilitation and Sports Medicine, 2^nd^ Faculty of Medicine, Charles University and University Hospital Motol, Prague, Czech Republic;

^3^Paraple Centre, Prague, Czech Republic

**Additional Methods:**

*Videos demonstrating the assessment of each grade of MES according to the rating algorithm*

Video 1

Response to exteroceptive stimuli

Left leg – no response

Right leg – squeezing the skinfold of the thigh and calf causes internal rotation in the hip joint

Response to proprioceptive stimuli

Left and right legs – no response (during maximal flexion, a subtle movement of another leg is caused by a change of pelvic position)

Conclusion: left leg – MES 0, right leg – MES 1

Video 2

Response to exteroceptive stimuli

Left and right legs – no response to squeezing the skinfold of the thigh and calf

Response to proprioceptive stimuli

Left leg – ankle dorsiflexion after maximal leg flexion

Right leg – no response

Conclusion: left leg – MES 1, right leg – MES 0

Video 3

Response to exteroceptive stimuli

Left leg – squeezing the skinfold of the thigh and calf causes big toe extension

Right leg – squeezing the skinfold of the thigh causes toes extension and internal rotation in the hip joint

Response to proprioceptive stimuli

Left leg – ankle dorsiflexion and inversion during leg extension

Right leg – toes extension during the initiation of leg movement and big toe flexion during leg flexion

Conclusion: left leg – MES 2, right leg – MES 2

Video 4

Response to exteroceptive stimuli

Left leg – squeezing the skinfold of the calf causes a complex motor reaction of the left leg and a weaker motor reaction of the right leg (less than one-third of the range of motion)

Right leg – no response to squeezing the skinfold of the thigh and calf

Response to proprioceptive stimuli

Left leg – ankle inversion and big toe extension during leg extension (catch during hip extension due to hip flexors spasticity)

Right leg – ankle dorsiflexion during leg flexion and complex motor reaction during leg extension

Conclusion: left leg – MES 3, right leg – MES 1

Video 5

Response to exteroceptive stimuli

Left and right legs – semi-flexion, internal rotation, and adduction in the hip joints to all stimuli

Response to proprioceptive stimuli

During passive movement of both legs, a strong motor response is presented in another leg, trunk, and both hands. Extensor spasm in the examined leg is shown during extension.

Conclusion: left leg – MES 4, right leg – MES 4

**Additional Results:**

A total of 50 participants were enrolled to the study. The individual participant data can be found in eTable 1.

| Participant | | | | | | | First Exam | | | | | | | | | | Second Exam | | | |  |  |
| --- | --- | --- | --- | --- | --- | --- | --- | --- | --- | --- | --- | --- | --- | --- | --- | --- | --- | --- | --- | --- | --- | --- |
|  | | | | | | |  | | Rater A | | | | Rater B | | | |  | | | |  |  |
| Personal Data | | | | | ISNCSCI | | PSFS | | MES | | MAS | | MES | | MAS | | MES | | MAS | | Order of Exam |  |
| ID | Sex | Age | Interval | Cause of Injury | NLI | AIS | Freq. | Sev. | R | L | R | L | R | L | R | L | R | L | R | L |  | Medication |
| 1 | M | 37 | 9 | Diving into water | C5 | B | 4 | 3 | 3 | 3 | 4 | 4 | 3 | 3 | 3 | 4 | 3 | 3 | 2 | 2 | ABB | AS |
| 2 | M | 48 | 21 | Traffic accident | C8 | A | 2 | 1 | 1 | 2 | 0 | 0 | 1 | 2 | 1 | 1 | 2 | 2 | 1 | 1 | BAB | 0 |
| 3 | F | 38 | 18 | Fall | T3 | A | 2 | 1 | 2 | 2 | 0 | 0 | 1 | 0 | 0 | 0 | 2 | 2 | 1 | 1 | BAA | 0 |
| 4 | M | 20 | 1 | Traffic accident | C8 | B | 2 | 1 | 1 | 2 | 1 | 1 | 2 | 2 | 1 | 1 | 2 | 2 | 1 | 1 | ABA | AS |
| 5 | M | 24 | 2 | Sport injury | C5 | A | 2 | 2 | 3 | 3 | 3 | 3 | 1 | 1 | 3 | 3 | 2 | 1 | 1 | 1 | ABA | AS |
| 6 | M | 49 | 7 | Sport injury | C3 | A | 2 | 2 | 2 | 2 | 3 | 3 | 4 | 4 | 2 | 2 | 2 | 2 | 2 | 2 | BAB | AS, AE |
| 7 | M | 40 | 2 | Diving into water | C4 | A | 2 | 1 | 2 | 2 | 1 | 1 | 1 | 1 | 0 | 0 | 2 | 2 | 1 | 1 | ABB | 0 |
| 8 | M | 42 | 4 | Fall | T6 | A | 1 | 1 | 2 | 2 | 0 | 1+ | 2 | 2 | 1 | 1+ | 2 | 2 | 0 | 0 | BAA | 0 |
| 9 | M | 31 | 6 | Traffic accident | C4 | A | 1 | 2 | 2 | 2 | 0 | 1 | 2 | 2 | 1 | 1 | 2 | 2 | 0 | 0 | ABA | AS, AE |
| 10 | M | 50 | 32 | Diving into water | C6 | A | 3 | 3 | 2 | 3 | 1 | 1 | 3 | 2 | 1+ | 2 | 2 | 2 | 1 | 1 | BAB | 0 |
| 11 | M | 60 | 15 | Fall | T4 | A | 2 | 1 | 2 | 2 | 0 | 0 | 2 | 2 | 0 | 0 | 2 | 2 | 0 | 0 | ABB | AE |
| 12 | M | 27 | 4 | Diving into water | C6 | B | 3 | 1 | 3 | 2 | 4 | 3 | 2 | 2 | 4 | 3 | 2 | 2 | 4 | 4 | ABA | 0 |
| 13 | M | 27 | 8 | Traffic accident | C5 | A | 3 | 2 | 2 | 2 | 1+ | 1+ | 1 | 2 | 1 | 1+ | 1 | 1 | 0 | 0 | ABB | 0 |
| 14 | M | 31 | 6 | Diving into water | C4 | A | 2 | 1 | 2 | 2 | 0 | 0 | 2 | 1 | 1+ | 1+ | 2 | 0 | 1 | 0 | BAB | 0 |
| 15 | M | 37 | 15 | Traffic accident | C7 | A | 2 | 1 | 1 | 2 | 1 | 1 | 2 | 2 | 1+ | 1+ | 2 | 2 | 1 | 1 | BAA | 0 |
| 16 | F | 39 | 12 | Sport injury | T5 | A | 1 | 1 | 2 | 2 | 1 | 1 | 2 | 1 | 0 | 0 | 2 | 2 | 0 | 0 | ABA | AE |
| 17 | M | 49 | 17 | Fall | C4 | A | 2 | 1 | 1 | 0 | 0 | 0 | 2 | 0 | 1+ | 0 | 2 | 1 | 1 | 1 | BAA | 0 |
| 18 | M | 27 | 5 | Traffic accident | C4 | B | 4 | 2 | 2 | 3 | 4 | 2 | 2 | 4 | 4 | 4 | 2 | 2 | 4 | 2 | ABA | AS |
| 19 | M | 18 | 2 | Diving into water | C4 | A | 2 | 3 | 2 | 2 | 2 | 2 | 2 | 2 | 2 | 2 | 2 | 2 | 1 | 1+ | ABB | AS, AE |
| 20 | M | 45 | 20 | Traffic accident | C6 | A | 3 | 2 | 2 | 2 | 0 | 0 | 1 | 2 | 1+ | 1+ | 2 | 2 | 0 | 1 | BAB | AS |
| 21 | M | 57 | 7 | Fall | C6 | A | 2 | 2 | 2 | 2 | 0 | 0 | 1 | 2 | 0 | 0 | 2 | 2 | 0 | 0 | BAA | AS |
| 22 | M | 38 | 21 | Traffic accident | C7 | B | 1 | 1 | 2 | 2 | 1 | 1 | 2 | 2 | 2 | 1 | 2 | 1 | 0 | 1 | ABA | 0 |
| 23 | M | 24 | 1 | Diving into water | C5 | B | 1 | 2 | 3 | 3 | 2 | 2 | 2 | 2 | 1 | 1 | 2 | 3 | 2 | 2 | ABA | AS |
| 24 | M | 19 | 1 | Sport injury | C6 | B | 3 | 2 | 4 | 4 | 4 | 4 | 3 | 3 | 3 | 3 | 2 | 2 | 3 | 4 | BAA | AS, AE |
| 25 | M | 44 | 19 | Sport injury | C5 | B | 1 | 1 | 2 | 2 | 1+ | 1+ | 2 | 2 | 2 | 3 | 2 | 2 | 0 | 1 | BAB | AS, AE |
| 26 | M | 28 | 3 | Fall | C7 | A | 2 | 1 | 1 | 1 | 0 | 0 | 2 | 1 | 1+ | 1+ | 1 | 1 | 0 | 0 | BAB | 0 |
| 27 | F | 30 | 7 | Fall | C4 | A | 2 | 2 | 1 | 2 | 0 | 0 | 2 | 2 | 1+ | 0 | 2 | 2 | 0 | 0 | ABB | AS |
| 28 | M | 68 | 18 | Violance | T11 | A | 1 | 1 | 1 | 1 | 0 | 0 | 2 | 2 | 1 | 0 | 2 | 2 | 1 | 0 | ABA | 0 |
| 29 | M | 18 | 2 | Sport injury | C6 | A | 2 | 2 | 2 | 2 | 1 | 1 | 2 | 2 | 2 | 2 | 2 | 2 | 3 | 4 | BAA | AS, AE |
| 30 | M | 47 | 21 | Fall | C5 | B | 3 | 3 | 3 | 1 | 4 | 1 | 4 | 3 | 3 | 3 | 2 | 2 | 2 | 1 | BAA | 0 |
| 31 | M | 18 | 2 | Traffic accident | C4 | A | 2 | 2 | 2 | 3 | 1+ | 1+ | 4 | 2 | 2 | 1+ | 3 | 3 | 1 | 1 | ABA | AS, AE |
| 32 | M | 39 | 16 | Sport injury | C4 | B | 3 | 2 | 3 | 3 | 3 | 2 | 3 | 3 | 1 | 1+ | 4 | 3 | 2 | 1 | ABB | AS |
| 33 | F | 37 | 2 | Infection | C4 | A | 3 | 3 | 2 | 3 | 4 | 4 | 3 | 3 | 3 | 4 | 4 | 2 | 4 | 4 | ABA | AS, AE |
| 34 | M | 42 | 2 | Fall | C8 | A | 3 | 2 | 2 | 2 | 1+ | 3 | 2 | 2 | 1 | 1+ | 3 | 2 | 1 | 1+ | BAB | AS |
| 35 | M | 34 | 3 | Fall | T6 | A | 4 | 3 | 4 | 4 | 2 | 4 | 4 | 4 | 3 | 3 | 4 | 4 | 3 | 4 | BAB | AS, AE |
| 36 | M | 18 | 1 | Sport injury | C4 | B | 2 | 1 | 4 | 3 | 1+ | 2 | 3 | 3 | 1 | 2 | 3 | 3 | 1 | 2 | BAB | AS, AE |
| 37 | F | 20 | 1 | Traffic accident | T6 | B | 1 | 2 | 3 | 3 | 4 | 3 | 3 | 3 | 2 | 3 | 3 | 2 | 4 | 4 | BAA | AS, AE |
| 38 | M | 42 | 12 | Traffic accident | T7 | A | 2 | 2 | 2 | 2 | 1 | 1 | 3 | 2 | 1+ | 1 | 2 | 3 | 1 | 2 | BAA | 0 |
| 39 | M | 42 | 7 | Traffic accident | C6 | C | 4 | 3 | 3 | 3 | 4 | 4 | 3 | 3 | 3 | 3 | 3 | 2 | 4 | 4 | BAA | 0 |
| 40 | M | 37 | 11 | Sport injury | T4 | A | 3 | 2 | 4 | 3 | 4 | 1 | 3 | 3 | 2 | 1 | 3 | 3 | 3 | 1+ | BAB | 0 |
| 41 | M | 29 | 7 | Traffic accident | C8 | B | 2 | 3 | 2 | 3 | 0 | 1 | 2 | 3 | 1+ | 2 | 1 | 3 | 1 | 1 | BAA | AS |
| 42 | F | 53 | 15 | Fall | T7 | A | 2 | 3 | 4 | 3 | 2 | 1+ | 3 | 3 | 2 | 2 | 4 | 3 | 2 | 1+ | BAA | 0 |
| 43 | M | 36 | 17 | Fall | T7 | A | 1 | 1 | 4 | 3 | 1+ | 2 | 3 | 3 | 4 | 3 | 2 | 3 | 4 | 3 | ABA | 0 |
| 44 | M | 27 | 10 | Diving into water | C5 | A | 2 | 3 | 4 | 4 | 2 | 2 | 4 | 2 | 4 | 3 | 4 | 3 | 2 | 1+ | BAA | AS |
| 45 | M | 45 | 1 | Fall | T9 | A | 3 | 2 | 2 | 2 | 3 | 3 | 3 | 2 | 4 | 3 | 2 | 2 | 3 | 3 | ABB | 0 |
| 46 | M | 28 | 1 | Sport injury | T4 | B | 1 | 2 | 2 | 3 | 4 | 2 | 2 | 2 | 3 | 3 | 4 | 2 | 4 | 3 | BAB | AS |
| 47 | M | 32 | 3 | Traffic accident | C5 | A | 2 | 2 | 2 | 3 | 3 | 2 | 3 | 4 | 2 | 3 | 3 | 4 | 2 | 3 | BAB | AS, AE |
| 48 | M | 44 | 1 | Traffic accident | T2 | A | 2 | 2 | 4 | 3 | 1+ | 1 | 3 | 4 | 4 | 3 | 2 | 2 | 2 | 1+ | BAA | AE |
| 49 | F | 28 | 1 | Diving into water | T2 | A | 2 | 3 | 2 | 2 | 3 | 3 | 2 | 2 | 1+ | 2 | 2 | 2 | 1+ | 1+ | BAA | 0 |
| 50 | F | 56 | 1 | Fall | C4 | A | 4 | 2 | 2 | 2 | 3 | 3 | 3 | 3 | 3 | 3 | 3 | 2 | 3 | 3 | BAB | AS, AE |

Abbreviation: ID, participant ID; Interval, time from injury; ISNCSCI, International Standards for Neurological Classification of Spinal Cord Injury; NLI, Neurological Level of Injury; AIS, ASIA Impairment Scale; PSFS, Penn Spasm Frequency Scale; Freq., Frequency; Sev., Severity; MES, Muscle Excitability Scale; MAS, Modified Ashworth Scale; AS, Antispastic; AE, Antiepileptic
